# Sleep Fragmentation, Not Nocturnal Hypoxemia, Is the Primary Correlate of Attentional Slowing in Obstructive Sleep Apnea

**DOI:** 10.3390/jpm16020117

**Published:** 2026-02-14

**Authors:** Márcio Luciano de Souza Bezerra, Sergio Luis Schmidt, Eelco van Duinkerken, Andreza Maia, Ana Luiza Caldas Coutinho, Kai-Uwe Lewandrowski

**Affiliations:** 1 Post-Graduate Program in Neurology, Hospital Universitário Gaffrée e Guinle, Universidade Federal do Estado do Rio de Janeiro, Rio de Janeiro 20270-004, Brazil; marciolucianodesouzabezerra.be@gmail.com (M.L.d.S.B.); sergio.schmidt@unirio.br (S.L.S.); e.vanduinkerken@amsterdamumc.nl (E.v.D.); andreza.maia80@gmail.com (A.M.); analu.coutinho@edu.unirio.br (A.L.C.C.); 2 Department of Medical Psychology, Amsterdam University Medical Centers, Location Vrije Universiteit, 1081 HV Amsterdam, The Netherlands; 3 Division of Personalized Pain Research and Education, Center for Advanced Spine Care of Southern Arizona, Tucson, AZ 85712, USA; 4 Department of Orthopedic Surgery, University of Arizona Tucson Campus, Tucson, AZ 85724, USA

**Keywords:** obstructive sleep apnea, precision sleep medicine, personalized medicine, sleep fragmentation, sleep-stage instability, cognitive vulnerability, attention, phenotyping, endotypes

## Abstract

**Background:** Obstructive sleep apnea (OSA) is associated with slower response speed, yet conventional severity classification based on the apnea–hypopnea index (AHI) shows limited ability to predict cognitive outcomes. The AHI aggregates distinct pathophysiological processes, including intermittent hypoxemia and sleep fragmentation. Within emerging precision sleep medicine frameworks, disentangling these mechanisms is critical for improved phenotyping and personalized risk assessment. This study aimed to replicate prior findings using a Go/No-Go Continuous Visual Attention Test (CVAT) and to identify the most informative polysomnographic predictor of attentional performance in OSA. **Methods:** In this cross-sectional study, participants underwent full-night type I polysomnography and the CVAT. After exclusions, 84 patients with OSA and 22 polysomnographically normal controls were analyzed. The sample sizes for mean differences and correlational analyses were adequate. Attentional performance was indexed by standardized reaction time (RT), referenced to a normative database (n = 1244). Within the OSA group, linear regression with backward elimination evaluated hypoxemia and sleep fragmentation metrics. **Results:** Patients with OSA demonstrated significantly slower RTs than controls (*p* = 0.005). Within OSA, the AHI was not associated with attentional performance (*p* = 0.398). In the final regression model, sleep stage shifts—reflecting sleep–wake instability—emerged as the sole independent predictor of attentional slowing (β = 0.27, *p* = 0.013), whereas all hypoxemia indices were excluded. **Conclusions:** Sleep stage instability represents a cognitive vulnerability marker in OSA, independent of respiratory events. Integrating fragmentation metrics into precision sleep medicine models may enhance individualized phenotyping, identify patients at higher neurocognitive risk, and inform targeted interventions focused on stabilizing sleep architecture rather than relying solely on the AHI.

## 1. Introduction

Global estimates indicate that obstructive sleep apnea (OSA) affects nearly one billion adults worldwide, with prevalence ranging from ~9% to 38% depending on diagnostic criteria [1]. OSA is characterized by recurrent upper-airway collapse, resulting in intermittent hypoxemia and sleep fragmentation that disrupts restorative stages such as slow-wave and REM sleep [2]. Obstructive sleep apnea is conventionally diagnosed and classified in severity based on the apnea–hypopnea index (AHI) [3]. However, the AHI may not entirely reflect the physiological burden of OSA, including desaturation depth and duration, as well as sleep fragmentation [4].

Previous studies have reported the presence of cognitive deficits in OSA patients [5,6]. Attention is a core cognitive domain, since the other domains depend on attentional performance [7]. Consistent with limitations of the AHI, the association of the AHI with at-tentional deficits has been weak or even absent [8,9]. These findings suggest that the physiological consequences of OSA (desaturation and sleep fragmentation) may exert a more direct influence on attention dysfunction.

Indeed, evidence from multiple modalities indicates that OSA is consistently associated with attentional alterations, as highlighted by the recent systematic review on MRI data [10]. Specifically, previous research has shown that individuals affected by this condition exhibit extended reaction time (RT) [9]. However, these studies were conducted with a small sample size of OSA patients and controls. Additionally, the controls did not undergo polysomnography type 1 to rule out sleep disorders, and the data on RT were not standardized.

Based on this context, the present study had two main objectives. The first objective was to compare attentional performance between a larger sample of patients with OSA and sleep-disorder-free controls, verified by full-night type 1 polysomnography, using a 15 min Go/No-Go task (Continuous Visual Attention Test—CVAT), as well as stand-ardized measures of RT. Regarding this objective, we hypothesized that our group of people with OSA would show longer reaction times in comparison with controls. The second objective was to clarify the strength of the association between RT—as an index of vigilance—and markers of sleep fragmentation and/or nocturnal hypoxemia beyond conventional severity metrics such as the AHI. Following the previously observed lack of correlation between the AHI and reaction time in OSA [9], it might be hypothesized that sleep fragmentation may play a role in attention over desaturation.

## 2. Materials and Methods

### 2.1. Design

This was an observational cross-sectional study.

### 2.2. Participants

Between December 2021 and January 2025, outpatients undergoing type 1 polysomnography (PSG) at a private Sleep Medicine Service (RIOSONO) in Rio de Janeiro, Brazil, were asked to participate in this study. Of these, 207 consented. Eligible participants were aged between 18 and 60 and could present with or without sleep-related complaints.

A semi-structured clinical interview was conducted to screen for exclusion criteria, which included current or past history of alcohol or drug abuse, use of psychotropic medication, neurological (stroke, traumatic brain injury, epilepsy) or psychiatric conditions, pulmonary or neuromuscular disorders, and pregnancy. All participants underwent the 15 min version of the CVAT before PSG. Following the overnight PSG, the Epworth Sleepiness Scale and the Hamilton Depression and Anxiety Rating Scales were completed.

Two hundred and seven adults underwent full-night, type 1 polysomnography and completed a 15 min Go/No-Go Continuous Visual Attention Test (CVAT). Based on PSG results, after exclusion of other primary sleep disorders (e.g., severe insomnia, narcolepsy), 84 individuals with an AHI ≥ 5 events/h were classified as having OSA and included in the patient group, and 22 individuals with normal PSG findings were included in the control group. The remaining 101 participants were excluded due to other sleep disorders or not meeting specific inclusion/exclusion criteria for the study groups.

### 2.3. Institutional Setting

This study was conducted at RIOSONO in close collaboration with the Federal University of the State of Rio de Janeiro.

### 2.4. Ethical Consideration and Data Protection

The study was approved by the Ethics Committee of the Federal University of the State of Rio de Janeiro (CAAE: 69406817.1.0000.5258) on 2 December 2021. All participants provided written informed consent (Declaration of Helsinki). Data collection was fully anonymized based on subject number and saved on secure servers that were part of RIOSONO.

### 2.5. Sample Size Calculation

For objective 1, sample size was determined for a two-sided comparison of means between two independent groups, assuming a type I error rate (α) of 0.05 and 80% power. Clinically meaningful differences were defined a priori based on normative data, participant age and sex distributions, and published reference values. Under these assumptions, analyses for each parameter of the Continuous Visual Attention Test (CVAT) indicated that a minimum of 11 participants per group were required to detect the specified mean differences, assuming equal group sizes. Because unequal group enrolment was anticipated, statistical power was preserved by increasing the total sample size according to the expected allocation ratio. Assuming a 4:1 allocation ratio, a total sample size of 42 participants (14 in the smaller group and 28 in the larger group) was required to maintain 80% power. Sample size calculations were based on unadjusted comparisons of group means. Age and sex were included as covariates in the primary analyses to improve precision and account for potential confounding. No additional inflation of the sample size was applied, as covariate adjustment is expected to reduce residual variance.

For the regression analyses (Objective 2), sample size adequacy considering six candidate predictors, standard recommendations suggest a minimum of 10 outcome events per predictor to ensure stable model estimation. Therefore, a sample size with 84 and 6 predictors is adequate. To reduce the risk of overfitting, predictors were selected based on a priori clinical relevance, and results should be interpreted with caution.

### 2.6. Polysomnography and Parameter Quantification

Full-night assisted polysomnography was performed using sleep and monitored with digital polysomnography (BNT 36/BNT Poli; Emsamed, Rio de Janeiro, Brazil), with comprehensive monitoring including standard electroencephalographic (C3-A2, C4-A1, O1-A2, O2-A1), electro-oculographic, and electromyographic (submental and bilateral tibialis anterior) leads, supplemented by respiratory measurements (nasal pressure transducer, oronasal thermistor, thoracic/abdominal strain belts) and continuous pulse oximetry with body position monitoring. Obstructive sleep apnea was diagnosed with an apnea–hypopnea index (AHI) ≥5 events/hour according to the 2012 American Academy of Sleep Medicine criteria [3], defining apnea as ≥90% airflow cessation for ≥10 s and hypopnea as ≥30% airflow reduction with ≥3% oxygen desaturation or arousal. Oxygen desaturation parameters included event frequency (D1, dips ≥ 3%), desaturation index (D2), duration of hypoxemia (D3, SpO_2_ < 90%), and basal saturation (D4). Sleep fragmentation was assessed using the microarousal index (F1); sleep stage shifts (F2), which were operationally defined as transitions between different sleep stages occurring between consecutive 30 s epochs and sustained for at least one full epoch (≥30 s), in accordance with standard AASM scoring rules [3] and previous approaches to quantify sleep continuity [11,12]; wakefulness after sleep onset (F3); and the sleep fragmentation index (F4). We quantified the sleep fragmentation index (SFI) as the total number of awakenings/shifts to Stage 1 (from deeper non-rapid eye movement [NREM] or REM sleep) divided by the total sleep time in hours [13]. Sleep efficiency was calculated as the total sleep time relative to the time in bed, minus the sleep onset latency, with all analyses restricted to artifact-free periods.

### 2.7. Attention Assessment

**Figure 1:**
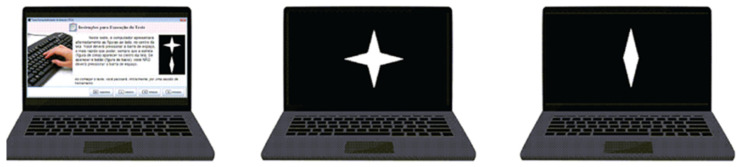
Schematic overview of the Continuous Visual Attention Test (CVAT). he test begins with on-screen instructions: “In this test, the computer will alternately display specific figures at the center of the screen. You must press the spacebar with your dominant hand as quickly as possible whenever the star appears in the center. If the diamond appears, you should not press the spacebar.” The target stimulus and non-target stimulus are each presented for 250 ms. The CVAT measures average reaction time (RT; mean reaction time for correct responses), intraindividual variability of reaction time (VRT; standard deviation of RTs across the test), omission errors (OE; failure to respond to targets), and commission errors (CE; responses to non-targets). English and Spanish versions of the test were made available upon request by Prof. Schmidt.

Participants completed the 15 min Computerized Visual Attention Test (CVAT, Figure 1), which has been extensively used in clinical research [14,15,16]. This test does not have a novelty effect because of the practice test subjects need to complete without error. Stability of attention effects can be measured with tests as short as 90 s, and a learning effect has been shown to not be present [14,15,16]. The test was administered on a Windows 10 laptop (13″ LCD display, <30 ms latency). Testing occurred in a quiet examination room with standardized conditions: 50 cm viewing distance and visual acuity ≥20/30 (corrected if needed).

The task required responses to target stimuli (star) among distractors (diamond). The paradigm consisted of 6 blocks (3 sub-blocks each, 20 trials/sub-block) with randomized interstimulus intervals (1, 2, or 4 s). Stimuli appeared for 250 ms. Primary outcomes were omission errors (OE), commission errors (CE), reaction time of correct responses (RT), and variability in reaction time (VRT—standard deviation of individual reaction times of correct responses). Participants were instructed to press the space bar as quickly and accurately as possible with the dominant hand.

### 2.8. Reference Group

Next, the raw CVAT parameters were standardized based on age and sex using a normative sample of 1244 individuals who completed the 15 min CVAT. The raw scores were normalized to rule out the effects of age and sex.

The reference group consisted of a subsample of individuals who underwent mandatory medical and psychological assessments for certification of fitness to drive between December 2021 and January 2025 in Brazil. These assessments were conducted by certified physicians and psychologists according to national regulatory standards. All individuals who presented for assessment were invited to participate in a national normative study involving the CVAT. Consenting participants completed the 15 min version of the CVAT on the same day and at the same location as their health examination for obtaining a driver’s license. A total of 1244 individuals who met the general inclusion criteria and passed the mandatory driving fitness assessment were included in the reference group. Eligibility required a normal neurological examination, intact visual and auditory function, and Mini-Mental State Examination scores within normative limits adjusted for educational level. Furthermore, all subjects scored above the 25th percentile on validated psychometric measures of intelligence quotient, global attention, and memory.

### 2.9. Statistical Analysis

Demographic and PSG variables were compared using a t-test for independent samples or a chi-squared test, where appropriate. The study was divided into two parts.

First objective: A univariate analysis of covariance (ANCOVA) was conducted with raw reaction time (RT) as the dependent variable, group (OSA vs. control) as the fixed factor, and age and sex as covariates. To confirm the results, we also performed an ANOVA using age- and sex-adjusted Z-scores for reaction time, derived from a normative database of 1244 healthy individuals. A Pearson correlation was calculated between the AHI and reaction time (RT) within the OSA group to assess whether the severity of sleep-disordered breathing predicted cognitive slowing.

Second objective: Secondly, within the OSA group, the association between normalized reaction time and the measures of desaturation and sleep fragmentation derived from polysomnography. For this, all measures of desaturation (D1–D4) and sleep fragmentation (F1–F4) were included in a backward regression model. To avoid multicollinearity, first, all variables were forced into the model, and those with a variance inflation factor (VIF) of ≥5 were excluded from the backward model to reduce redundancy. Variable entry and removal were based on the F-probability criterion (0.05 and 0.10, respectively).

## 3. Results

### 3.1. Demographic and Clinical Characteristics

As shown in Table 1, individuals with OSA were predominantly male and had higher BMI compared to comparison group (*p* = 0.003 and *p* < 0.001, respectively). We did not find significant group differences in age or subjective sleepiness (Epworth Sleepiness Scale; all *p* > 0.05). The AHI was significantly higher in the OSA group (*p* < 0.001), with a balanced number of mild, moderate, and severe OSA across the patient group. No significant differences were observed in total sleep time, sleep efficiency, or delta sleep (all *p* > 0.05) between the two groups. In contrast, REM sleep percentage was lower in OSA subjects (*p* = 0.042).

### 3.2. Desaturation and Sleep Fragmentation

As shown in Table 2, compared to the control group, participants with OSA exhibited significantly more severe nocturnal oxygen desaturation, as reflected by a higher number of desaturation events, an elevated oxygen desaturation index (ODI), and a longer cumulative time with SpO_2_ < 90% (all *p* < 0.01). Baseline oxygen saturation was also lower in the OSA group (*p* < 0.001). OSA subjects showed greater sleep fragmentation, with higher microarousal index and fragmentation index (both *p* < 0.001), and a modest increase in the number of sleep stage transitions (*p* = 0.046). Wake after sleep onset (WASO) did not differ significantly between groups.

### 3.3. First Objective

The ANCOVA with raw mean reaction time revealed a statistically significant main effect of group on reaction time (F (1, 102) = 4.91, *p* = 0.029, partial η^2^ = 0.046), indicating that individuals with OSA had significantly slower RTs compared to the control group after controlling for age and sex. Neither age nor sex was a significant covariate (*p* = 0.106 and *p* = 0.678, respectively). The ANOVA with the mean reaction time normalized for age and sex indicated a significant effect of group (F (1, 104) = 8.30, *p* = 0.005, partial η^2^ = 0.074). These results confirm that the OSA group performed significantly worse than the comparison group, further supporting the robustness of the group difference.

No significant association between the AHI and normalized RT within the OSA group was found (r = 0.093, *p* = 0.398), replicating previous findings that suggest the severity of OSA, as measured by the AHI, does not predict reaction time performance within individuals with OSA.

### 3.4. Second Objective

The regression model, which included all desaturation measures (D1–D4) and sleep fragmentation indices (F1–F4), showed that event frequency (D1, number of desaturations ≥3%) and the desaturation index (D2) exhibited VIF values above 5 but below 10, indicating moderate collinearity. These variables were therefore removed during the backward linear regression procedure, and all remaining predictors showed VIF values below 4. In the final model, only sleep stage instability (F2) remained significant. Higher sleep stage instability was associated with longer normalized reaction time, reflecting worse intrinsic alertness (F (1, 82) = 6.512, *p* = 0.013, R = 0.271, *p* = 0.013).

**Figure 2:**
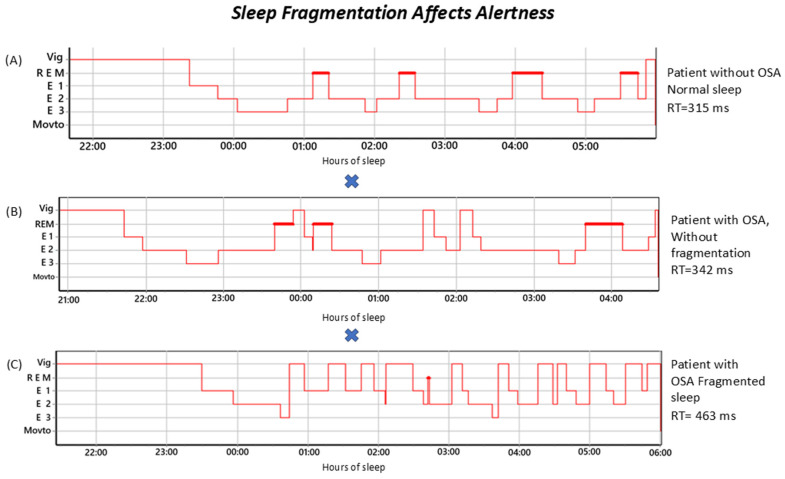
Effects of sleep fragmentation on reaction time in patients with and without obstructive sleep apnea (OSA). Note: Panel (**A**) shows a hypnogram of a patient without OSA, with normal sleep: 51-year-old male, AHI = 2.0 events/h, RT = 315 ms. (NORMAL). Panel (**B**) illustrates a hypnogram of a patient with OSA, without fragmentation: 36-year-old female, AIH = 23.4 events/h, RT = 342 ms. (NORMAL). Panel (**C**) depicts a hypnogram of a patient with OSA, with fragmented sleep: 32-year-old male, AHI = 50.0 events/h, RT = 463 ms. (|z| > 1.64). RT = reaction time; Vig = wakefulness; REM = rapid eye movement; E1 = N1 (Stage 1); E2 = N2 (Stage 2); E3 = N3 (Stage 3); Movto = movement. Bold red lines represent rem sleep stage.

The hypnograms in Figure 2 show examples of a control subject, one OSA participant without fragmentation, and one OSA participant with fragmentation. This illustrates that, despite elevated AHI values, no clear correlation is observed between the apnea–hypopnea index and alertness, indicating that the AHI alone does not capture the degree of attentional impairment associated with prolonged reaction times.

## 4. Discussion

This study confirms the results of our previous study, which showed that individuals with OSA exhibit significantly slower reaction times compared to rigorously verified, sleep-disorder-free controls, even after adjusting for age and sex using z-scores referenced to a large normative dataset. The AHI showed no association with attentional performance, highlighting the limited value of this traditional severity marker in predicting cognitive impairment. Of all polysomnography-derived measures, only sleep stage shifts correlated with slower RT. These results indicate that attentional deficits in OSA are dissociable from respiratory event frequency and are likely driven by sleep fragmentation.

The present finding of an increase in RT using another larger OSA sample and a control group that had Type 1 PSG demonstrated that our previous study [9] is stable and reliable. Moreover, the use of Z-scores from a large normative sample gives further support for the reliability of our results. Although the control group included a higher proportion of women, who typically exhibit slower reaction times than males [15], the OSA group, which was predominantly male, still showed significantly longer reaction times. This pattern suggests that slowing is more strongly driven by OSA-related mechanisms than by sex differences. In fact, the overrepresentation of women in the control group may have introduced a conservative bias, potentially increasing mean reaction times in that group and reducing the between-group contrast, thereby further reinforcing the robustness of our findings.

Our results further indicate that sleep continuity and microarchitecture are key factors in reaction time (RT) impairment associated with OSA. This result supports previous studies linking microstructural alterations (e.g., compound action potential rate, spindle dynamics) to cognitive dysfunction [16], as well as evidence that measures based on sleep continuity better predict cognitive gains related to CPAP use than nocturnal hypoxemia [17,18]. While several associations reached statistical significance, the observed effect sizes were modest, suggesting that sleep fragmentation represents one contributing factor—rather than a dominant or exclusive determinant—of attentional slowing in OSA. From a personalized medicine perspective, this underscores the multifactorial nature of cognitive vulnerability in OSA and highlights the need to integrate sleep fragmentation with additional biological, behavioral, and clinical markers to improve individualized risk stratification.

The finding of an absence of association between desaturation indices and slowness in OSA is in contrast with the results of Kainulainen et al. [5]. These authors used a refined metric of hypoxic load and found that this metric was associated with slow responses in the Psychomotor Vigilance Task (PVT) [5]. Even though we did not calculate this metric, it is important to consider that the PVT selectively requires stimulus-guided vigilance, which may be particularly susceptible to hypoxemia-related fluctuations in alertness. In contrast, the Go/No-Go paradigm used in the present investigation requires sustained monitoring and descending inhibitory control, in addition to motor speed. Neuroimaging data indicate that these partially dissociable attentional networks are modulated by sleep stage stability, suggesting that fragmentation acts through pathways extending beyond transient alertness failures [19].

In the multivariate context, when physiological markers are evaluated concurrently, conventional desaturation indices lose their predictive value for attentional performance, whereas sleep stage shifts retain independent explanatory significance. This pattern supports the interpretation that disruption of stable sleep continuity represents a meaningful pathological mechanism rather than a statistical artefact. In a Go/No-Go paradigm, reaction time slowing should therefore not be regarded as mere psychomotor delay; instead, it reflects reduced efficiency of sustained monitoring and top-down inhibitory control—attentional processes that are particularly sensitive to fragmentation-related instability in sleep architecture.

From a mechanistic perspective, recurrent transitions between non-REM (NREM) and REM (rapid eye movement) sleep are known to disrupt autonomic balance [20] and thalamo-cortical organization [21], both of which are crucial for maintaining attentional networks. Furthermore, instability in sleep stages is postulated to compromise the neurodependent mechanism of “neuronal pumping” described by Jiang-Xie et al. [22]. In this mechanism, synchronized neuronal activation drives waves of interstitial fluid essential for glymphatic clearance. Disruption of neuronal synchrony due to fragmentation can negatively affect this process, leading to decreased metabolic clearance. Consequently, instability in sleep architecture may be directly linked to attentional functioning.

Sleep stage shifts have emerged as a clinically relevant marker of attentional vulnerability in OSA. Beyond indicating non-restorative sleep, repeated shifts in vigilance states trigger sympathetic surges and cardiometabolic strain. Remarkedly, arousal burden has been independently associated with hypertension, regardless of apnea severity [23], supporting fragmentation as an active pathological driver rather than merely a consequence of respiratory events. Interventions that enhance sleep continuity—through optimized CPAP adherence, behavioral strategies, or targeted adjunctive therapies—may therefore provide cognitive benefits that exceed those achieved by AHI reduction alone. Despite this clinical relevance, fragmentation metrics such as sleep stage shifts remain absent from most routine PSG reports. Incorporating them into standard evaluation may improve cognitive risk stratification and guide more mechanism-based management in OSA.

A few limitations warrant consideration. First, the cross-sectional design precludes causal inference. Secondly, residual confounding, such as genetic susceptibility or vulnerability to sleep disruption, cannot be excluded. Third, fragmentation metrics did not distinguish between respiratory-related and spontaneous arousals. As these arousal subtypes may arise from different physiological mechanisms (e.g., hypoxia/hypercapnia-driven versus cortical or autonomic instability), they could have distinct effects on sleep architecture and cognitive outcomes. Future studies using arousal-specific scoring, combined with high-resolution autonomic monitoring or event-locked EEG analyses, are needed to determine whether respiratory-mediated and spontaneous arousals contribute differently to attentional impairment in OSA. Fourth, high-resolution hypoxic indices, such as hypoxic load [5], were unavailable due to software constraints. Fifth, there is a sex imbalance between the two groups, with more male participants in the OSA group. As males are generally faster on reaction time tasks, it is unlikely that the longer reaction time in OSA is driven by the sex imbalance. Sixth, BMI is higher in the OSA group, which is a consequence of our inclusion criteria. We tested the correlation between BMI and reaction time in OSA (β = 0.133 *p* = 0.228) and controls (β = 0.163 *p* = 0.468). This indicates that, while BMI is important for OSA prevalence, it did not affect our reaction time results. Seventh, statistical approaches cannot fully prevent confounding. However, our analysis with age- and sex-matched z-values demonstrated similar results and the correlation with BMI were statistically non-significant. Therefore, it is unlikely that these factors affected our results. Eighth, we were unable to study other aspects of attention, such as divided attention or attentional switching. Finally, although sleep stage shifts showed independent predictive value, routine clinical implementation is limited by the lack of standardized automated scoring tools. Future research should develop automated microstructure metrics and use longitudinal designs to validate their clinical relevance.

Future research should also employ and interventional designs to overcome the limitation of cross-sectional studies that not allow for causal inference. Such studies, although hampered by the lack of standardized sleep fragmentation calculation methods, should examine whether targeting sleep stage instability yields improvements in processing efficiency and real-world functional outcomes. Integrating cognitive phenotyping with physiological subtyping may ultimately enable more personalized and clinically meaningful management of cognitive risk in OSA.

## 5. Conclusions

In conclusion, our first objective showed that the average reaction time was significantly increased in individuals with OSA, independent of AHI severity. Importantly, our second objective showed that sleep stage shifts, but not oxygen desaturation, predicted slower responses in a Go/No-Go Continuous Performance Test, identifying sleep continuity as a modifiable target for reducing cognitive deficits in OSA. These findings support an expansion of the current diagnostic paradigm for obstructive sleep apnea by complementing traditional respiration-centered metrics with sleep continuity-based risk markers that more accurately capture attentional deficits. Within a precision sleep medicine framework, the incorporation of sleep fragmentation measures can enhance individualized phenotyping, enable the identification of patients at increased neurocognitive risk, and inform targeted interventions aimed at stabilizing sleep architecture, rather than relying solely on AHI-based severity classification.

## Figures and Tables

**Table 1:** Demographic, clinical, and polysomnographic characteristics of OSA patients and control Subjects.

Variable	OSA (n = 84)	Controls (n = 22)	*p*	|Cohen’s d|
Sex, male/female (male %)	58/26 (69.0%)	7/15 (31.8%)	0.003	—
Age (years)	41.46 ± 1.19	37.41 ± 2.66	0.136	2.53
Body mass index (kg/m^2^)	30.50 ± 0.57	24.85 ± 0.67	<0.001	9.55
Epworth Sleepiness Scale (score)	10.24 ± 0.58	8.18 ± 1.07	0.106	2.91
Apnea–hypopnea index (events/h)	25.48 ± 2.04	2.41 ± 0.27	<0.001	12.63
Sleep apnea severity				
Mild (AHI 5.0–14.0)	27 (32.1%)	—	—	—
Moderate (AHI 15.0–29.0)	30 (35.7%)	—	—	—
Severe (AHI > 30.0)	27 (32.1%)	—	—	—
Total sleep time (min)	395.68 ± 5.24	381.50 ± 15.09	0.383	1.72
Sleep efficiency (%)	83.76 ± 0.97	81.18 ± 2.71	0.377	1.73
N1 + N2 sleep stage (% TST)	76.48 ± 1.14	72.16 ± 2.15	0.085	3.08
REM sleep stage (% TST)	8.86 ± 0.59	11.50 ± 1.13	0.042	3.61
Delta sleep (% change)	13.03 ± 0.69	16.24 ± 1.91	0.125	3.04
Delta sleep (min change)	47.63 ± 2.59	57.05 ± 6.56	0.123	2.51

Categorical variables are presented as n (%). AHI: apnea–hypopnea index; TST: total sleep time; REM: rapid eye movement. Em dashes indicate not applicable. OSA: obstructive sleep apnea.

**Table 2:** Comparative analysis of nocturnal oxygen desaturation metrics, sleep fragmentation parameters, and CVAT outcomes in OSA versus control subjects.

Variable	OSA	Controls	*p*
Desaturation variables			
Number of desaturations (D1)	85.54 ± 12.19	8.14 ± 2.83	<0.001
Oxygen desaturation index (ODI > 3%, events/h) (D2)	13.05 ± 1.81	1.54 ± 0.61	<0.001
Time with SpO_2_ < 90% (min) (D3)	5.93 ± 1.19	1.77 ± 0.73	0.004
Baseline SpO_2_ (%) (D4)	94.65 ± 0.19	95.68 ± 0.12	< 0.001
Fragmentation variables			
Microarousals (events/h) (F1)	31.51 ± 2.35	10.34 ± 1.38	<0.001
Sleep stage shifts (n) (F2)	29.13 ± 1.54	24.14 ± 1.90	0.046
Wake after sleep onset (WASO, min) (F3)	25.64 ± 2.78	32.89 ± 6.17	0.293
Sleep fragmentation index (F4)	59.66 ± 4.54	23.18 ± 2.29	<0.001
CVAT variables			
Reaction time, correct responses (ms)	425.23 ± 5.05	396.09 ± 7.19	0.007
Variability of reaction times, correct responses (ms)	88.79 ± 3.28	92.55 ± 6.42	0.602
Coefficient of variability of reaction times, correct responses	0.21 ± 0.01	0.23 ± 0.01	0.154

Note: Data are presented as mean ± standard error of the mean. These analyses were corrected for age and sex. OSA = obstructive sleep apnea; CVAT = Continuous Visual Attention Test.

## Data Availability

The original contributions presented in this study are included in the article. Further inquiries can be directed to the corresponding author.
